# A Review of the Academic and Psychological Impact of the Transition to Secondary Education

**DOI:** 10.3389/fpsyg.2018.01482

**Published:** 2018-08-29

**Authors:** Danielle Evans, Giulia A. Borriello, Andy P. Field

**Affiliations:** ^1^School of Psychology, University of Sussex, Brighton, United Kingdom; ^2^Department of Psychology, The Pennsylvania State University, University Park, PA, United States

**Keywords:** school transition, anxiety, psychological health, academic achievement, secondary education, primary education

## Abstract

The transition from primary to secondary education is one of the most stressful events in a young person’s life (Zeedyk et al., 2003) and can have a negative impact on psychological well-being and academic achievement. One explanation for these negative impacts is that the transition coincides with early adolescence, a period during which certain psychological disorders (i.e., anxiety disorders) become more salient (Kessler et al., 2005) and marked social, biological, and psychological development occurs (Anderson et al., 2000). This review evaluates the existing literature on the psychological and academic impacts of the transition to secondary education on young adolescents. We examine the factors that plausibly increase or mitigate the risk of developing mental health issues and/or a decline in academic performance during the transition to secondary education. We also review the interplay between psychological health and academic achievement across and beyond the transition. We conclude with a summary of what schools and parents can learn from these findings to support children in a successful transition into secondary education.

## Introduction

The transition from primary to secondary education is a normative event for most children around the world, which typically occurs when children are early adolescents (mostly between the ages of 10–14). Although most students change school at some point during their education, systems around the world vary significantly. For example, in England, children transition in Year 6 at age 11, whereas in the United States (US), the age and grade of transition differs per school and per state, with children transitioning between the ages of 10 and 14 to a middle or high school (5th and 8th grade, respectively). While it is the norm to transition, it is possible that children may also attend schools in which they complete their education in one institution, though these are uncommon in the United Kingdom. To avoid switching between locale-specific terms, for the entirety of this review, primary education refers to schooling before children transition to a middle school, high-school (United Kingdom), secondary school, or a gymnasium around the ages of 10–14, while secondary education refers to schooling after this transition.

Around two in five students fail to reach their expected progress following the transition to secondary education (Galton et al., 1999), with around 40% of students making no progress in English and reading (42 and 38%, respectively) and 34% making no progress in maths from Year 6 (age 10–11) to Year 7 (age 11–12) (Galton et al., 1999). In the United Kingdom, Ofsted (2002) concluded there was limited preparation available for the differences in teaching and learning children face after the transition.

The transition to secondary education has received increased interest from researchers in recent years, with many researchers regarding the change as one of the most stressful events young adolescents will experience (Chung et al., 1998; Coelho and Romão, 2016). Children report additional concerns during this time, including fear of bullies, being lost, peer relationship worries, and anxiety over coping with an increased workload (Zeedyk et al., 2003). Additionally, the transition to secondary education can directly impact educational attainment, with a reported interruption in students’ academic growth during the transition year (Akos et al., 2015).

**FIGURE 1:**
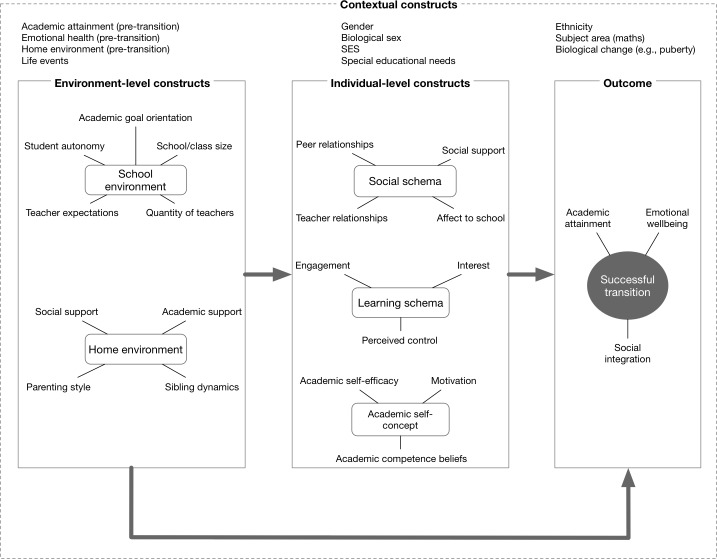
Summary of the key constructs influencing a successful transition to secondary education.

**Figure 1** shows an attempt to organize the various constructs that, based on research, contribute to a successful transition to secondary education. The first consideration is what is deemed a “successful” transition. Although the adjustment to secondary education can be measured in various ways, most researchers regard it to encompass social, academic, and emotional adaptation (Duchesne et al., 2012). Hall and DiPerna (2017) particularly note the importance of relationships with peers, developing academic abilities, and a stable state of mental health as vital components of adjusting to secondary education. These components are not independent. Where there are declines in emotional well-being, there are also declines in peer relationship quality, and academic performance, though causality has yet to be established (e.g., Woodward and Fergusson, 2000; Reijntjes et al., 2010; Mundy et al., 2017; Rahman et al., 2018). Although many students adapt with relatively few issues, others find the transition impacts one, two, or all of these domains. It has been argued that children who express more worries prior to the transition are less likely to be well-adjusted in all three of these areas (Duchesne et al., 2012).

In terms of what predicts a successful transition, **Figure 1** organizes the key constructs into higher-order categories of contextual, environment-, and individual-level constructs. Contextual constructs are variables that could plausibly moderate any of the relationships between the environmental-level constructs, individual-level constructs and a successful transition. The contextual constructs could all plausibly have a direct impact on whether the transition is successful too. They have in common that they are either fixed during transition (e.g., pre-transition academic attainment/emotional health, biological sex, whether we consider the model for a specific subject area such as maths), likely not to change (e.g., SES), or the effect of change is likely to be fixed at the group level (for example, we might consider the effects of puberty to be somewhat similar for all boys). The environment- and individual-level constructs are ones that are highly likely to change heterogeneously during transition to secondary education (for example, there is likely to be considerable variability in how dramatically the school environment changes for different children). They differ, self-evidently, in whether they relate to the child’s environment or his/her internal schema.

Within these categories, we have pulled together related measures from the literature into superordinate constructs. For example, researchers have looked at class size, teacher expectations, and academic goal orientation as predictors of a successful transition, all of which can logically be grouped as part of the school environment. Similarly, a child’s set of beliefs about their social environment and her/his emotional responses to them (which we have labeled *social schema*) encompasses peer relations, teacher relations, affect to school (or belongingness), and social support more generally. The constructs identified are not exhaustive or definitive, they are merely a convenient way to organize the existing literature for the purpose of this review.

Within the environment-level constructs, most of the literature relates to changes in the child’s school or home environment. The overall school environment typically changes during the transition to secondary education. Children often move from a smaller, personal primary school where they are taught by a single teacher in, primarily, a single classroom, to a larger, more complex, impersonal secondary school where they attend lessons in different locations with different teachers, often with larger class sizes. Secondary school buildings tend to be larger, and individuals are often required to travel further afield, often on public transport.

Within the individual-level constructs, the literature focuses on the belief systems in the child about their social position, their learning, and their academic self-concept. For example, Cantin and Boivin (2004) report a decrease in friendship network size following the transition, meaning children have fewer friends post-transition. Similarly, Martínez et al. (2011) note a decline in both general social support and the support given by teachers at this time. These findings are represented by social schema in **Figure 1**.

The constructs identified are, of course, not independent. The environment-level constructs could plausibly have a direct effect on a successful school transition, but also an indirect effect by influencing any one of the individual-level constructs. For example, the classroom goal structure alters post-transition with higher importance placed on performance goals, where the focus is on demonstrating ability relative to others, as opposed to mastery goals, where the focus is on increasing competence relative to self-set standards (Madjar et al., 2018). This shift in goals in turn negatively impacts social schema such as school belongingness/engagement (Madjar and Chohat, 2017). Similarly, decreased emotional support in the classroom has been reported following the transition (Shell et al., 2014), which is likely to impact social schema. The individual-level constructs are also likely to influence each other: social and learning schemas are both likely to influence academic self-concept and plausibly each other.

We aim to evaluate the academic and psychological impact of the secondary education transition, while examining risk and protective factors that may amplify or lessen these effects. Based on Hall and DiPerna’s (2017) definition, we primarily review the evidence concerning emotional well-being and academic performance. Because so little is known about the causal relationships between the identified constructs we believe it is misleading (not to mention messy) to make individual connections in **Figure 1** between the various constructs that might imply causality. Instead, this review will highlight the relationships between the constructs in the **Figure 1** that have been observed, but there may be other connections that, as yet, have not been explored empirically.

## The Effects of the Transition to Secondary Education on Academic Achievement

The first indicator of a successful transition to secondary education is academic achievement (**Figure 1**). Academic achievement is essential for individual well-being across the lifespan (Gottfredson, 2004; Fiscella and Kitzman, 2009). The primary-to-secondary transition is a critical period of development in which many children are particularly vulnerable to lower levels of academic achievement. Low academic achievement during early adolescence is linked with various negative consequences, including early pregnancy and higher delinquency rates (Kasen et al., 1998; Freudenberg and Ruglis, 2007; Henry et al., 2012). Moreover, low achievement during this period tends to be succeeded by school dropout and low occupational achievement and income across the lifespan (Day and Newburger, 2002). In this section, we summarize research that has examined the impacts of the primary-to-secondary transition on academic-related outcomes in early adolescence.

A number of United States studies indicate that the transition from primary to secondary education has a negative impact on student grade point averages (GPA) and academic achievement (Felner et al., 1981; Simmons et al., 1991; Seidman et al., 1994; Alspaugh and Harting, 1995; Gutman and Midgley, 2000; Dotterer et al., 2009). Illustratively, United States students who moved from a primary to a secondary school experienced a decline in grades following the transition, unlike students who were in the same grade but had not transitioned to secondary education (Felner et al., 1981). Alspaugh (1998) also found that U.S. children experience lower academic achievement after transitioning from primary school to secondary school. Moreover, children who transitioned into secondary education where peers attended a variety of primary schools experienced lower levels of achievement than children who transitioned into secondary education with peers who attended the same primary school. Results from achievement at the high school level indicated that U.S. children who transitioned multiple times, from primary to secondary school and from secondary school to high school, experienced higher declines in achievement than those who had fewer school transitions. Alspaugh’s (1998) work is in line with other evidence (Rice, 2001) suggesting that the transition from primary to secondary education can have long-term negative consequences on academic outcomes.

### Environment-Level Constructs

With respect to the environment-level constructs in **Figure 1**, developmental psychologists have attempted to understand why and how the transition to secondary education can negatively affect academic achievement. The *stage-environment fit* model (Eccles et al., 1993) suggests that a mismatch between children’s developmental needs at the time of the transition and the social context of secondary schools contributes to a decline in academic outcomes following the transition.

Several aspects of the secondary education school environment that differ from primary education may have a particular effect on academic achievement following the transition; these include new academic environments (e.g., new, larger schools and classrooms) and different structural demands (e.g., switching classrooms, teachers, and classroom materials for each subject throughout the day). Children must also forge new student-teacher relationships and adjust to changes in teacher expectations and declines in student autonomy.

Although teachers have more control over secondary education classrooms, academic standards tend to be higher in secondary education than in primary, and require more intrinsic motivation from adolescents (Harter et al., 1992). Compared to primary education, secondary education classes place an increased emphasis on grades and teachers’ academic expectations of students tend to be higher (Eccles and Midgley, 1989; Wigfield et al., 1991). Moreover, children may perceive classroom goals differently in primary and secondary education. Cross-sectional work indicates that in primary education settings, students report being more *task oriented*, or engaged in academic work for the sake of learning, whereas in secondary education settings children report being more *performance oriented*, or engaged in academic work for the sake of demonstrating ability (Anderman and Midgley, 1997). A longitudinal study found that following a transition from primary to secondary education, children perceived classroom climates to focus more on competence and less on learning (Anderman and Midgley, 1997). These changes subsequently influence individual-level constructs such as adolescents’ academic self-concept, interest and engagement (learning schema), and affect toward school (social schema), as discussed in the following sections.

### Individual-Level Constructs

#### Academic Self-Concept

Academic self-concept, or self-perceptions regarding academic topics and learning, has various components, including a cognitive component specific to academic competence and an affective-motivational component (Marsh et al., 1991; Arens et al., 2013a). Studies indicate that academic self-concept decreases between the end of primary and the beginning of secondary education (Wigfield et al., 1991; Arens et al., 2013a). For example, Coelho et al. (2017) found decreases in students’ academic self-concept from their last year in primary education to the end of their first year in secondary education, along with lower levels of self-esteem. Although this sample included Portuguese children who transitioned to secondary education 2 years earlier than students in the United Kingdom or the U.S. typically do, these findings are consistent with studies examining children who transition to secondary education at a later age (Wigfield et al., 1991). In addition, Seidman et al. (1994) found that students’ academic self-perceptions declined even after adjusting for student age, grade level, and ability level. Together, these findings provide evidence that the transition process itself (i.e., the environment) as well as individual factors (e.g., developmental changes), likely play a role in changing children’s academic self-concept.

However, there is inconsistency in the literature with not all studies finding a decline in perceived academic competence after the transition to secondary education. For example, Harter et al. (1992) found no significant differences between children’s perceptions of scholastic competence following the transition to secondary education compared to children who did not transition. There are also studies demonstrating *increases*, not decreases, in academic self-efficacy (Zimmerman and Martinez-Pons, 1990; Midgley et al., 1995). The variance in observed changes in perceptions of academic competence across the transition period must be explained by other factors, including how much children value a particular academic domain and their interest in it. Moreover, we might expect changes in academic self-concept to differ by discipline. For example, Wigfield et al. (1991) found significant declines in children’s perceived competence in English following the transition to secondary education, but only marginal declines in mathematics. In the Section “Learning Schema,” we discuss student attitudes and interest in a select few academic domains.

#### Learning Schema

Students tend to hold more negative attitudes toward certain academic domains including mathematics and science, compared to others, and to academic achievement, more broadly (Eccles et al., 1984). Student self-perceptions about their own abilities in and attitudes toward maths and science tend to decrease as children progress in school, and especially during the transition to secondary education (Eccles et al., 1984; Midgley et al., 1989a). For example, a cross-sectional study by Barth et al. (2011) found that children’s attitudes toward and self-efficacy in mathematics and science declined during the transition. Similarly, student interest in mathematics and science were lower after, rather than prior to, the transition to secondary education. Furthermore, Australian students reported less involvement in the classroom and declining enjoyment and attitudes toward maths following the transition relative to those yet to make the move over to secondary education (Deieso and Fraser, 2018).

Differences in learning experiences surrounding science and mathematics before and after the primary-to-secondary transition may also influence changes in students’ academic attitudes and interests. Prior to the transition, for example, students do not have a choice in the type of science or mathematics courses in which they enroll and instruction in these arenas is standardized across students. Following the transition to secondary education, students have more agency in the number and type of mathematics and science courses they choose. Moreover, student ability in these domains becomes more salient, and students tend to get grouped into courses with students who have similar abilities to their own. Moreover, as children progress through school, teacher support decreases. Barth et al. (2011) investigated whether the role of teaching effectiveness and student perceptions of positive teaching strategies (i.e., teacher support, engaging instruction) contributed to decreases in student interest and attitudes toward maths and science. Findings indicated that effective teaching and student perceptions of positive teaching strategies strongly predicted changes in student interest and self-efficacy in mathematics and science, particularly during the transition to secondary education. Thus, one way that negative attitudes toward and low self-efficacy and interest in mathematics and science across the transition period may be counteracted is via teacher support.

The transition to secondary education also affects engagement and perceived control in learning. A study by Rudolph et al. (2001) examined whether student perceptions of control over their academic outcomes as well as student investments in academic success influenced their ability to successfully transition from primary to secondary education. The researchers expected to find that high perceptions of control and personal investment in academic success would encourage academic engagement and ultimately, academic achievement. Conversely, they expected that low levels of control and investment would promote academic disengagement and hinder academic achievement. Results indicated that, compared to students who did not transition from primary to secondary education, students who experienced a transition and reported lower levels of perceptions of control and personal investment in school also reported higher levels of stress and depressive symptoms. The authors suggest that students with lower levels of perceived control over and personal investment in academic pursuits are more likely to become disengaged from school and to find it easier to feel overwhelmed or particularly sensitive to any school-related issues.

#### Social Schema

Student feelings of “belongingness” at school and how much they enjoy school may also be impacted by school transitions and affect child achievement. Because intervention research suggests that a positive school climate can benefit children’s mental health and academic outcomes, several studies have investigated whether changes in the school climate between primary and secondary education contributes to declines in academic outcomes post-transition (Battistich et al., 2004). Illustratively, Riglin et al. (2013) examined bidirectional associations between young adolescents’ (*M*_age_ = 11.78 years) affect toward school and their academic achievement using a prospective, longitudinal design. They found reciprocal associations between school liking and academic achievement at the beginning and end of the first year of secondary education. However, after controlling for conduct problems, degree of liking school predicted later academic achievement, but early achievement no longer predicted later school liking. These findings support the notion that affect toward school and a sense of belonging to school are linked with academic achievement (Resnick et al., 1997; Roeser et al., 2000; McLaughlin and Clarke, 2010). However, some studies have not found evidence to suggest that children’s perceptions of the school climate differ prior to and following the school transition (Thornburg and Glider, 1984; Fenzel and Blyth, 1986; Hirsch and Rapkin, 1987; Crockett et al., 1989; Harter et al., 1992), and other studies report positive child perceptions of the school climate post-transition (Schulenberg et al., 1984; Nottelmann, 1987; Berndt and Mekos, 1995). These findings indicate a need for more research to investigate links between the school climate before and after the primary-to-secondary education transition and its effects on academic achievement in early adolescence and beyond.

#### Interplay Between Individual-Level Constructs

It is important to understand the interplay between academic achievement, social schema, learning schema, and academic self-concept because doing so provides clear target areas to help children to maintain academic achievement across the transition to secondary education. The way that students cope with changes in academic achievement after the transition may have long-term consequences for future achievement. If initial decreases in grades or achievement post-transition leads some students to alter their academic self-concept, they may become more disengaged with school and have more negative feelings toward school, or increased feelings of disconnectedness. In turn, teachers may interact with students in a more negative way and these effects can snowball and lead to future decreases in student achievement or engagement (Eccles et al., 1993; Fenzel, 2000). Conversely, it is also possible that for students with more resources and personal investments in learning, initial declines in grades following the transition may motivate them to become more engaged at school and work harder to bring up their grades. Thus, the ability of a young adolescent to continue doing well academically following a school transition is likely to depend on both the interplay between the individual-level factors and the environment-level factors that influence them.

### Contextual Variables

So far, we have reviewed the environment- and individual-level factors affecting the transition from primary to secondary education. However, a range of contextual variables have been identified as predictors of academic performance across the transition to secondary education. For example, pubertal status has been linked to changes in academic self-concept and self-representation, both of which are important for academic achievement (Schaffhuser et al., 2017). This section summarizes what we know about some of the contextual variables identified in **Figure 1**.

#### Gender

Few studies examining the school transition have reported consistent evidence of gender differences impacting future academic outcomes (Wigfield et al., 1991; Harter et al., 1992; Seidman et al., 1994). For example, Seidman et al. (1994) found that children’s grades declined following the transition regardless of gender. Studies examining motivation, attitudes toward, and self-concepts in specific academic domains have found that boys tend to have more positive attitudes toward and higher self-concepts in maths than girls, whereas girls tend to have more positive attitudes toward and higher self-concept in English than boys (Eccles et al., 1984; Marsh, 1989). However, research findings regarding effects of gender on self-concepts and attitudes toward academic achievement, and how these characteristics vary by gender across the transition period, are inconsistent.

#### Cognitive and Emotional Traits

Studies have reported a number of cognitive or emotional traits that influenced findings regarding the school transition and academic outcomes (Petrides et al., 2004). For example, a longitudinal study examined the development of self-control during early adolescence, as children transitioned from primary to secondary education, and found that students with higher levels of self-control adjusted better following a school transition, receiving higher grades in English, maths, and science courses (Ng-Knight et al., 2016).

Several studies suggest that how well children adapt to a new school environment and perform academically may depend on ability level in academic domains prior to a transition. For example, Wigfield et al. (1991) examined students’ academic self-concept in mathematics prior to and following a secondary education transition, and found that children’s mathematics self-concept following a school transition varied by level of mathematics ability. For students with high mathematics ability, mathematics self-concepts declined over time following the transition, while students with low mathematics ability experienced slight increases in their mathematics self-concepts following the transition. Other studies have also reported that academic ability level can help explain effects of school transitions on young adolescents’ academic-related outcomes (Midgley et al., 1989a,b; Anderman, 1998). For example, in a longitudinal study (Midgley et al., 1989a) teacher influences on student perceptions of the importance of mathematics before and after the secondary transition depended on student mathematics achievement. Results indicated that, compared to high achieving students, low achieving students had steeper declines in perceptions of mathematics importance if they switched from more supportive teachers before the transition to less supportive teachers following the transition.

With respect to emotional traits, Qualter et al. (2007) found that compared to students with below average emotional intelligence, those with average or higher levels of emotional intelligence received better grades in school, and had fewer teacher concerns regarding effort following the transition to secondary education (age 11–12).

#### SES and Ethnicity

Because youth of lower socioeconomic status (SES) tend to have lower academic achievement than higher SES youth (McLoyd, 1998), it is possible that the primary-to-secondary education transition is especially stressful for this group of children. Moreover, because far more ethnic/racial minorities tend to live in poverty (Brooks-Gunn et al., 1996), it is also pertinent to consider how the transition may impact ethnic and racial minorities’ academic achievement. Illustratively, Simmons et al. (1991) found that grades of African American students were extremely low following the secondary education transition, even though all students grades declined. Serbin et al. (2013) investigated academic achievement across the secondary transition in an “at risk” sample of children from lower income families. Findings indicated that family resources and child gender mattered: children from families with fewer resources had lower achievement than those from families with more resources following the transition, and girls had higher grades than boys following the transition. Moreover, multiple mediation analyses demonstrated that the link between gender and achievement was mediated by children’s social and academic skills (i.e., spelling), as well as the degree of support they received from parents prior to the transition. Thus, social skills, academic skills, and support from parents prior to the transition contributed to differences in boys’ and girls’ achievement following the transition into secondary education. Future work should focus on prevention and intervention efforts for populations of children that may especially need help to navigate the secondary education transition and to excel in school.

## The Effects of the Transition to Secondary Education on Emotional Health

The second key indicator of a successful transition to secondary education in **Figure 1** is emotional health. Adolescence is a significant period for the development of mental health disorders with symptoms often increasing during this time (e.g., Kessler et al., 2005). A report by the Office for National Statistics states the prevalence of mental health disorders to be 12% in children aged 11–16, compared to 8% of those aged 5–10 (see Green et al., 2005). The Australian National Survey of Mental Health and Well-Being supports this figure reporting that at least 14% of adolescents younger than 18 were diagnosable with a mental disorder (Sawyer et al., 2001). Given the high frequency of disorders within this age range, it seems likely that the primary-to-secondary education transition could contribute to mental health issues among young adolescents. In this section, we review this possibility.

### What Do We Mean by Emotional Health?

If we consider emotional health in terms of constructs identified by mental health practitioners (APA, 2017a), then there are broadly two categories of symptom clusters to consider: symptoms that are largely internal to the person (manifest in psychological constructs such as anxiety and depression) and those that are external to the person (manifest in constructs such as conduct problems and attention-deficit and hyperactivity). Before looking at predictors of these symptoms related to the transition to secondary education, we will review these symptom clusters.

#### Internalizing Symptoms

One of the most common childhood disorders is anxiety. Anxiety disorders can take many forms and are generally characterized as feelings of tension and worrisome thoughts as well as physiological changes including an increased heart rate, increased perspiration and trembling among others (APA, 2017a). Anxiety is reported to be the earliest disorder to emerge in childhood, with 50% of anxiety disorders beginning by age 6 in affected adolescents (Merikangas et al., 2010). Additionally, when averaged across all subtypes of anxiety disorders, the median age of onset is 11 years (Kessler et al., 2005). Furthermore, it is one of the most common disorders faced by children; one meta-analysis of 41 studies spanning 27 countries conducted between 1985 and 2012 estimated the worldwide prevalence of any anxiety disorder to be 6.5% (Polanczyk et al., 2015). Moreover, the lifetime prevalence of any anxiety disorder appears to be a staggering 31.9% (Merikangas et al., 2010). Childhood anxiety also has a higher prevalence than depression, and is diagnosed more frequently than behavioral issues such as conduct disorder (Cartwright-Hatton et al., 2006).

Most anxiety disorders are already established by early adolescence (Kessler et al., 2005) with little change in frequency from age 13/14 up to age 17/18 (Merikangas et al., 2010). The transition to secondary education typically occurs just before this period of a child’s life and is arguably particularly important in this process. For example, students experiencing greater worries concerning the school environment and relationships over the transition typically have heightened anxiety symptoms (Arowosafe and Irvin, 1992; Harter et al., 1992; Lucey and Reay, 2000; Akos and Galassi, 2004). Greater school transition concerns both prior to and following the move have been associated with increased anxiety (Rice et al., 2011), though research to date has been somewhat sparse and inconsistent.

A longitudinal study of U.S. schoolchildren aged 11–13, showed a decrease in anxiety symptoms following the move to secondary education (Grills-Taquechel et al., 2010). This decrease in social anxiety was significant only in males. Furthermore, anxiety symptoms were predicted by global self-worth and social acceptance, with higher levels of both predicting greater decreases in anxiety, with males again showing greater declines. This finding suggests the transition could be particularly beneficial for male students in reducing their anxiety. One reason proposed to explain this gender difference is that females tend to participate in “relational” forms of bullying such as gossiping, spreading rumors, and excluding peers (Crick and Grotpeter, 1995; Murray-Close et al., 2007). This may be an underlying mechanism of why girls experience greater social anxiety compared to boys, given females place greater value on close friendships, and greater fear of rejection and the loss of relationships (for a review, see Rose and Rudolph, 2006). Additionally, Grills-Taquechel et al. (2010) reported lessened impact of the transition on individuals with high self-worth and those who felt more socially accepted by their peers. Meanwhile, there also appears to be links between anxiety, stress, and the transition. Zandstra et al. (2015) reported a negative transition experience was associated with declines in mental health well-being, evident only in individuals with high awakening cortisol, a hormone important for stress reactivity. This association suggests that some individuals may be predisposed to greater emotional responses following a negative event such as the secondary education transition. This may help to explain why some individuals transition successfully, while others do not.

In a Canadian cohort of 11-year-old pupils, Duchesne et al. (2009) found that anxiety predicted both academic and teacher worries preceding the transition to middle school. Further analysis suggests girls perceived themselves as being more anxious and also reported greater worries about meeting academic demands and establishing relationships with teachers. However, attachment predicted anxiety levels, with individuals reporting more ‘secure’ attachments showing lower anxiety levels. Alternatively, another study of over 200 English schoolchildren (age 11) found similar levels of anxiety both at the start of secondary education and at the end of their first year (Riglin et al., 2013). There were further gender differences with females experiencing higher general anxiety and school anxiety, greater school concerns, and increased school engagement compared to males, whereas, conduct disorder was higher in males than females. However, there were no measures prior to the transition in this study (e.g., in Year 6) making it impossible to draw conclusions about the effect of the transition.

Where evidence is inconsistent concerning the emergence of general anxiety following the transition to secondary education, there are links between the changeover and the development of one domain-specific type of anxiety: maths anxiety. Maths anxiety is often defined as feelings of tension, apprehension, or fear that may interfere with maths performance (Ashcraft, 2002). Maths anxiety has been found to increase at the time of the changeover for students that transitioned to a new secondary school compared to those that did not, and increased especially in females and high-achievers (Madjar et al., 2016). Their analysis suggests there is a significant increase in maths anxiety toward to end of primary education, which remains high for some time, before decreasing at the end of their first year in secondary education back to initial levels. This suggests that the transition to secondary education may be an important period for interventions for these groups because maths anxiety has been linked to GPA and maths ability (Madjar et al., 2016).

A second kind of internalizing disorder is depression. Depression is closely associated with anxiety that manifests in several symptoms most commonly including feelings of sadness, lack of interest and pleasure in activities, lack of energy and concentration, feelings of worthlessness, and recurrent thoughts of suicide (APA, 2017b). The prevalence of major depressive disorder (MDD) by age 14 is 8.4% (Merikangas et al., 2010), and a figure that almost doubles to 15.4% from age 13–14 to age 17–18 suggesting that adolescence is a critical period for developing depression. Further support for this argument comes from reported increases in suicidal ideation at a similar age to the transition to secondary education (Adrian et al., 2016).

Similar to the evidence regarding the transition to secondary education and anxiety symptoms, research on depression in this context is also somewhat sparse. Nevertheless, depressive symptomology is highly stable throughout adolescence, however, stability significantly drops during the transition from 6th to 7th grade coinciding with the transition from primary to secondary education (Tram and Cole, 2006). Rice et al. (2011) further report positive associations between depression and post-transition school concerns.

Additionally, in a study of over 2000 Scottish pupils, West et al. (2010) reported that poorer transitions at age 11 (including both school and peer concerns, such as increased workload and bullying) predicted depression at age 13 and 15, while peer concerns at age 13 was weakly associated with psychological distress at age 18 (OR = 1.19). Their results contradict Kingery et al. (2011) data showing depression significantly decreases following the transition. However, the sample in Kingery et al.’ (2011) study included mostly Caucasian children residing in small, rural, suburban communities which may make generalizability to children studying in urban districts, or those from ethnically diverse backgrounds, problematic.

While the conclusion is somewhat unclear, depressive symptoms are important to keep in mind because Riglin et al. (2013) reported greater levels of depression at age 11 predicted academic achievement at the end of individuals’ first year of secondary education. However, when controlling for conduct disorder, this effect was no longer significant. Additional gender effects were evident with depression being significantly associated with poorer academic achievement for males only. The mediating effect of conduct disorder between depression and achievement further highlights that emotional problems and academic achievement are not independent outcomes, and that declines in one area often coincides with declines in other domains.

Although the evidence linking the transition to secondary education to mental health outcomes is sparse, there is a larger body of research linking it to psychological attributes such as self-esteem, self-efficacy, and self-concept (e.g., Coelho et al., 2017). Though there are slight differences in meaning, these variables can be broadly defined as attitudes, beliefs, and models of a person’s own abilities, and their capability to perform such behaviors in a given situation (APA, 2017c). These concepts may be informative with respect to mental health outcomes because they have been linked to well-being, academic achievement, and other educational benefits (e.g., Diener and Diener, 1995; Marsh and Craven, 2006).

In a study of over 1100 Portuguese students, Coelho et al. (2017) reported decreases in academic self-concept, physical self-concept, and self-esteem after the primary-secondary education transition. The effect remained significant when controlling for gender. However, the school transition occurs slightly earlier in Portugal: as young as 9 years old. Additionally, self-esteem has been found to decrease during transitionary years and continues to decline post-transition (Seidman et al., 2003; Arens et al., 2013b). This decline is supported by Schaffhuser et al. (2017) who also reported decreases in self-esteem as well as academic and behavioral self-concepts over the transition. Moreover, student self-efficacy appears to be positively associated with teacher-rated overall school adjustment, as well as pupil-rated post-transition relationships with teachers (Bailey and Baines, 2012). In addition, West et al. (2010) indicated self-esteem may act as a predictor of adjustment, with individuals low in self-esteem prior to the transfer experiencing a poorer transition to secondary education with greater school and peer concerns. Despite a general consistency in the transition having a negative effect on self-esteem, Kingery et al. (2011) report the opposite: self-esteem *increased* following the transition.

To summarize, the evidence concerning the impact of the transition on psychological outcomes has been inconsistent. While some researchers have concluded the transition is detrimental to emotional well-being and psychological attributes, others disagree. One consistent finding across domains is the effect of the transition is heightened for individuals expressing greater concerns before the changeover. It appears that adolescents who express more worries regarding the transition are more likely to suffer poorer transitions compared to their peers. In addition, compared to boys, girls have been reported to experience a poorer transition, with heightened levels of anxiety, and greater concerns over relationships and workload (e.g., Duchesne et al., 2009; Riglin et al., 2013). In the Section “Externalizing Symptoms and Anti-Social Behaviors,” we describe a separate category of emotional health symptoms, externalizing symptoms, and predictors of these symptoms that relate to the transition to secondary education.

#### Externalizing Symptoms and Anti-social Behaviors

Externalizing disorders can include a range of disruptive behaviors including conduct disorder, aggression, attention deficit hyperactivity disorder (ADHD), and oppositional behavior. Although such issues can often be disruptive to the learning environment in the classroom itself, they are also related to negative outcomes for the individual including low achievement, school dropout, and non-completion of further education (Adams et al., 1999; McLeod and Kaiser, 2004; Reid et al., 2004; Finn et al., 2008).

There is a lack of research examining externalizing disorders and anti-social behaviors resulting from the primary-secondary education transition, although evidence suggests that the transition is an important event that may exacerbate the effects of externalizing disorders on educational outcomes. For example, Palmu et al. (2017) reported associations between conduct disorder and ADHD on academic performance during the transition to secondary education. In a study of over 300 12- to 13-year-old pupils in Finland, their results found externalizing behaviors were associated with a decrease in GPA, particularly, ADHD before the transition negatively affected GPA post-transition. Riglin et al. (2013) support this link with conduct problems prior to the transition associating with academic achievement post-transition. Additional analyses suggested that conduct problems pre-transition were also associated with a decrease in school liking following the move.

West et al. (2010) found higher levels of aggression were associated with poorer school transitions, but better peer transitions. Furthermore, aggressive behavior predicted academic expectations for secondary education as well as academic functioning post-transition (Cillessen and Mayeux, 2007). Aggression appeared to interact with peer status such that there was no significant effect of aggressive behavior on academic functioning in individuals with high popularity. Conversely, adolescents with low popularity and high aggressive behavior were more likely to experience lower academic functioning.

It is evident that there are individual differences. For example, three types of aggressive behavior trajectories have been identified over the transition including low-stable, decreasing, and increasing (Malti et al., 2015). Membership to a group was altered by the child’s views on friendships. Specifically, those who had less of an understanding of the value of trust and reciprocity within friendships were more likely to be in the increasing trajectory group. This highlights the importance of relationships with other individuals around the time of the school transition and may be a protecting factor against maladaptive outcomes.

### Predictors of Emotional Health

The review above demonstrates that the school transition impacts a wide range of emotional health outcomes and adolescent behaviors; however, the success of the transition can also be influenced by a number of other factors under the categories identified in **Figure 1**. These additional influences can increase or decrease the risk of a poor transition and include the individual’s social network (family, peers, and teachers), special educational needs (SEN), as well as gender and pubertal status. Generally, the children most at risk of poor transitions are children recognized as SEN and those with a poor social network.

#### Environment-Level Predictors

As children progress to secondary education, they often face a substantially different environment compared to the one that they have been used to. This environment includes larger classrooms and school buildings to navigate, and different social networks. The move to secondary education also leads to changes in the educational goal structure toward performance-based goals (Madjar et al., 2018). In a study of 415 schoolchildren (aged 11–12), Madjar et al. (2018) investigated goal orientations following the transition to secondary education. At present there are three commonly identified academic goals including: mastery-approach, which involves learning to acquire knowledge and skills; performance-approach, which involves demonstrating greater skills relative to others; and performance-avoidance, which involves avoiding demonstrating such skills. Madjar et al. (2018) argue that the transition from primary to secondary education coincides with a change from a mastery goal structure, to a performance goal structure post-transition. Further research provides evidence for performance goal structures being detrimental to school engagement (Madjar and Chohat, 2017). These results suggest that the school transition may increase competition between individuals, and not always for the better (see Johnson et al., 1981). Transitioning schools could focus more on continuing to provide a mastery goal structure given its importance for engagement, school performance, and general learning (see Kaplan and Maehr, 2007). Otherwise, this change in goal structure may increase the risk of school disengagement post-transition, which can already be a common issue as teens grow older.

Duineveld et al. (2017), who studied the secondary education transition in Finland, reported decreased depressive symptoms, decreased life satisfaction, and increased emotional exhaustion following the transition. In terms of the home environment, they found that mothers provided greater autonomy support (i.e., supporting the child’s self-governance and control over their life; Keller, 2016) compared to fathers prior to the transition. Moreover, Duineveld et al. (2017) reported greater levels of autonomy support before the transition significantly predicted a decline in depression after children moved to secondary education. This finding indicates that autonomous, supportive parenting that encourages independence may protect children from developing mental health disorders during the transition to secondary education.

Related evidence provides support for child-mother attachment predicting perceived academic competence and anxiety during the middle school transition (Maltais et al., 2017). The protective power of attachment has also been argued to moderate the relationship between a social comparison learning environment in the classroom on anxiety symptoms (Maltais et al., 2015). Furthermore, in a study of transitioning students, Booth-LaForce et al. (2012) reported that high-increasing growth in anxious withdrawal was predicted by low parental autonomy, low time spent with the mother, both restrictive and nurturing parenting, and peer exclusion. These findings further support the links between a positive, supportive social network, and adolescents’ behavioral outcomes during the transition.

#### Individual-Level Predictors

Social support is vital for early development, learning, and psychological well-being (e.g., Demaray and Malecki, 2002). Perceived social support can be very important during adolescence where individuals experience rapid changes biologically, emotionally, and socially. In addition, a positive social support network can also be protective of issues arising during the transition to secondary education. As we have mentioned above, parental support may buffer children from the emotional effects of the transition to secondary education (presumably through positive effects on the child’s social schema). The vast majority of research looking at the emotional impacts of the transition to secondary education has looked at variables that we have clustered as indicators of a child’s *social schema* in **Figure 1**.

First, peer relationships facilitate a positive transition on a range of adaptation measures. For example, Kingery et al. (2011) found that pre-transition positive peer relationships (e.g., peer acceptance, friendship quality, number of friends) predicted various positive post-transition well-being measures including academic achievement, loneliness (or lack of), self-esteem, and school involvement. Cantin and Boivin (2004) reported the transition to secondary education was associated with an increase of perceived social acceptance, as well as an increase in supportive relationships with their school friends. The increases reported were related to friends providing greater instrumental support, informational support, and emotional support. The effects remained in the following 2 years of school following the move. These findings further highlight the importance of friendships and peer relationships during the progression to secondary education.

In addition to the reorganization of children’s friendship networks, there is also evidence that the transition may provide new opportunities for victimized children. Wang et al. (2016) argued that victimization decreased for females following the school transition compared to girls that did not transition. Though, transition status made no difference in male victimization between the two time points. Additionally, exclusion and victimization have been argued to decrease following the transition on average, with those recognized as anxious-solitary youth experiencing greater relative declines (Shell et al., 2014). This idea supports the notion of secondary education providing a chance for children to alter their identity as a victim which they may have been associated with in primary school, and also provides the opportunity for children to find new friends or change social groups to one which is more positive and supportive. Cantin and Boivin (2004) also state that around 61% of school peer ties identified prior to the transition no longer remained following the move, further reinforcing the idea that children renegotiate their social network.

Overall, there is general support that a positive social network is important for children’s well-being during the transition to secondary education, but children also require support from their teachers and school to feel more secure in their new environment.

A second protective factor against negative emotional outcomes includes school connectedness and belongingness (*affect to school* in **Figure 1**). For example, Vaz et al. (2014a) studied 266 Australian pupils, and reported increases in school belongingness resulted in decreases in mental health problems, even when controlling for prior mental health. Moreover, in a comparison study between transitioning and non-transitioning schools in Australia and Denmark, respectively, Nielsen et al. (2017) reported no significant difference in school connectedness in transitioning schools as pupils aged, whereas schools that did not transition experienced significant decreases in school connectedness over time. However, it may be important to highlight that although the number of “disconnected” students in the transitioning sample was similar across all age groups, this was close to significance in the transition year with increased odds of disconnectedness. This means that, although not statistically significant, there was a trend toward disconnectedness increasing during the transition year.

A third aspect of social schema is the relationship to teachers. After the transition to secondary education, children tend to have different teachers for each discipline, compared to having a single teacher in primary education. It can be difficult for children to form relationships with their new teachers as strong as those held previously. This change in their social network may be detrimental given preadolescents’ need for guidance and support during this time (Eccles and Roeser, 2009). In addition, students have been found to possess a greater reliance on teacher support and a preference for external direction following the transition to secondary education (Robbers et al., 2018). This change in the type of relationship with teachers has been notably recognized as one of the concerns amongst children moving into secondary education (Duchesne et al., 2009).

Research findings concerning teacher relationships have been somewhat mixed. Martínez et al. (2011) describes how both social and teacher support significantly decline over the transition. Bru et al. (2010) also reported a general decline in perceived teacher support over the school years, nevertheless, they argue there is no obvious abrupt change during the time between primary and secondary education and criticize previous studies for not accounting for age-related differences. Despite mixed findings, teachers are likely to be an important part of the child’s social and support network, which we have seen is important for promoting well-being. Future research could therefore do more to look at the role of teachers within the child’s support network. For example, it would be beneficial to have research evaluating whether making student-teacher relationships and student-teacher support networks similar to those experienced in primary education leads to improved outcomes after the transition to secondary education.

#### Contextual Predictors

The previously described issues of declining social support and the different school climate are faced by every student. However, it is clear some students face additional difficulties when adjusting to secondary education, this includes children with SEN. SEN refers to children who have learning problems or disabilities that may make it difficult to learn relative to other children their age. This can include difficulties in reading and writing, behavioral issues, difficulty understanding or expressing themselves, as well as physical ability issues which may affect them while in school.

The school transition can be especially problematic for children with SEN. For children with sensory or mobility difficulties, solely moving between classes can be challenging, especially so in an unfamiliar environment such as a large, novel secondary school. Children with behavioral or emotional issues may have difficulty establishing relationships with teachers and peers, leaving them feeling isolated. With high importance placed on discipline and obedience to authority in secondary education, children with behavioral or emotional difficulties may be perceived by teachers as acting out or “troublemakers” when in fact they have different needs and requirements.

Fortunately, recent research has attempted to investigate the effects of the school transition more thoroughly for children with SEN, with the aim of identifying the most common issues and difficulties they may encounter during this change. In a systematic review examining the effects of the school transition for children with SEN, Hughes et al. (2013) investigated psychosocial functioning including internalizing functioning, self-concept, self-esteem, self-confidence, externalizing functioning, and social functioning. They identified key findings of a higher likelihood of victimization and bullying, poorer social adjustment (i.e., loneliness) and lower levels of perceived social support relative to typically developing children. Furthermore, children reported additional concerns and worries. These concerns referred to the provision for special needs in their new school, the ability to make friends, increased workload, and greater worries of bullying relative to their peers without SEN.

Since Hughes et al. (2013) conducted their review, a number of additional studies have been published finding similar results. First, Akos et al. (2015) reported less growth in both maths and reading during the transition year for SEN students despite having the largest year-to-year growth in the year prior to the changeover. Further research also reports individuals with disabilities display significantly lower academic competence compared to their typically developing peers pre- and post-transition (Vaz et al., 2014b). Though interestingly, adolescents with a disability showed an improvement in academic competence over the transition compared with other pupils. Conversely, disability status was linked to decreased mental health functioning pre- and post-transition. It is evident that adolescents with SEN have different requirements when moving to secondary education to ensure a successful transition. Neal et al. (2016) endorse a personal approach when designing transition strategies especially for children with SEN.

With respect to other potential contextual variables, gender has been found to predict adaptation to secondary education. This finding is not surprising given that (1) males and females develop at different rates during adolescence; and (2) internalizing disorders are more prevalent in females than to males (Kessler et al., 2005; Merikangas et al., 2010). Coelho and Romão (2016) found that females experienced significantly higher academic and peer-related stress during the school transition compared to their male peers, whereas males reported higher stress regarding teachers and rules. Furthermore, females had significantly greater increases in peer-related stress during the transition. Grills-Taquechel et al. (2010) supported this conclusion with results suggesting that males experienced a significant decrease in anxiety during the transition to secondary education whereas girls did not. In addition, girls experienced significantly greater general and school anxiety pre- and post-transition. Females also reported a higher number of school concerns, but greater school liking and fewer conduct problems pre- and post-transition (Riglin et al., 2013). Rice et al. (2011) also found school concerns were higher for females both prior to and following the transition. This finding is also supported by Smyth (2016) who reported that girls were more likely than boys to experience transition difficulties.

Furthermore, girls are more likely to experience maths anxiety over the transition to middle school. Madjar et al. (2016) found that girls reported higher maths anxiety following the transition to secondary education which later decreased to initial levels 1-year post-transition, whereas maths anxiety reported by boys remained stable during this time. This is an important finding as maths anxiety has been argued to have a bidirectional relationship with maths performance (Carey et al., 2015). Consequently, if maths anxiety increases for girls during this time it may also impact their later performance, which in turn may increase their anxiety toward maths. Schaffhuser et al. (2017) also argued females are more negatively impacted by the transition compared to males. However, it is not entirely negative for females whereby girls in fact report higher academic and social functioning post-transition relative to boys (Cillessen and Mayeux, 2007). On the other hand, Kingery et al. (2011) reported no gender differences in the overall adjustment to secondary education.

In addition, females tend to experience pubertal onset earlier than males (Lee, 1980) which can have interesting interactions with the effect of the school transition. As described above, females tend to experience greater stress levels around the transition which is also around the time of pubertal onset. Koenig and Gladstone (1998) reported higher rates of depression among developing females (i.e., those that had started or in the latter stages of pubertal development) during the transition years, whereas rates among males were stable over time. However, it is important to note that this was examined in a high school sample as opposed to the earlier transition of middle school/secondary education. By the time of the transition in this sample, a large number of females had already fully developed.

## Discussion

The aim of this review was to assimilate the findings to date concerning the impact of the primary-to-secondary education transition on both academic and psychological outcomes. Overall, there appears to be some negative impacts of the transition, though it is difficult to conclude definitively because there are many inconsistencies in the data. These conflicts are not unexpected given the multitude of interacting factors that exacerbate or mitigate the impact of the transition. However, there are still some findings worth noting.

Firstly, the transition to secondary education appears to have some negative consequences for academic achievement (Felner et al., 1981; Alspaugh and Harting, 1995; Alspaugh, 1998; Gutman and Midgley, 2000). Upon transitioning, students must adjust to larger schools and class sizes, greater academic independence, navigating new teacher and peer relationships, higher teacher expectations, and a bigger emphasis on grades and performance. These differences require children to adjust to new academic expectations, norms, and evaluation criteria. These differences can adversely impact young adolescents’ academic motivation and engagement, academic self-concept or competence, affect toward school and learning, and their intrinsic interest in school (Harter, 1981; Eccles et al., 1984; Eccles and Midgley, 1989; Skinner et al., 1998). Individual difference factors including cognitive and emotional ability levels, gender, and SES can moderate associations between these factors and future academic achievement following the secondary education transition.

With respect to children’s emotional health, the evidence was inconsistent. Some researchers found significant negative impacts on emotional well-being post-transition, while others found positive outcomes, or negligible results. Clearly, we need a better understanding of the interplay between the constructs identified in **Figure 1** to get a handle on what moderates the effect of the transition on emotional health.

Several risk and protecting factors were identified to play an important role in the transition. First, things likely to affect the child’s social schema were found to be particularly important over the transition, including social support received from parents, teachers, and peers. During the transition, children often renegotiate their friendship groups and report decreased general social support during this time. Cohen and Wills (1985) reviewed two main explanations for the role of social support during stressful situations: first, that social support acts as a buffer against stress and second, that solely being part of a social network is helpful for the individual. Their review found support for both explanations, suggesting social support provides various benefits during stressful events and daily life. These ideas may also support the evidence discussed in relation to the importance of social networks around the transition to secondary education. Due to the heightened stress felt by children during this time, social support may help them feel more secure and socially accepted. Parents and teachers should be made aware of the perceived decline in social support reported by adolescents and aim to provide additional support when required to allow for the best outcomes. Despite perceived declines in parental support and perceived increases in peer support during the transition, it is parental support that most accurately indicates emotional difficulties in adolescents (Helsen et al., 2000). Waters et al. (2014) support this finding, concluding that parental presence at home before and after school is the most significant predictor of a positive transition experience.

Additionally, the school and class environment can elicit negative outcomes. For example, children report higher performance-based goals in secondary education compared to the mastery approach in primary education. A performance-approach increases competition between individuals whereas a mastery approach focuses on learning and working with the purpose of gaining knowledge. This change can be harmful to engagement which is an important aspect to sustain during the transition.

Furthermore, some individuals are more “at-risk” of a poor transition relative to their peers. Those most affected include children with SEN. First, children with SEN may face additional difficulties during the move, including matters that may be seemingly straightforward such as transport and mobility, an issue that is usually not as applicable to typically developing children. Furthermore, the findings suggest children with SEN overall report higher victimization, poorer adjustment, lower levels of social support, and reduced academic growth during the transitional year. There are also noteworthy gender differences. The research suggests that females are arguably more affected by the transfer. Girls report higher rates of school concerns, as well as experiencing higher levels of anxiety and depression (in line with other research findings of higher rates of emotional and mood disorders in the adult population among females). Conversely, boys report higher concerns regarding rules and teachers, as well as higher rates of behavioral and conduct problems compared to females.

### Theoretical Explanations

Ideally, we need a theoretical model to explain how environment-level constructs create changes in the child’s individual-level schema, how environment-level constructs directly affect academic and emotional outcomes, and how individual-level schema affect academic and emotional outcomes. It is a tall order. There are too many potentially relevant theoretical frameworks to cover in one paper, but we can use the example of anxiety to look at how theories of emotion might prove useful.

First, we can look to these models to explain why the transition to secondary education might increase anxiety. Recent research has found very little evidence for the genetic transmission of anxiety, which implies that most risk comes from environmental transmission (Eley et al., 2015). Given that children spend a considerable proportion of their day in school, the school environment is likely to be a potential anxiety trigger. Learning theories suggest that anxiety is acquired through an association-based system in which stimuli and situations come to evoke fear through direct association with fear-evoking experiences, verbal threat information, and observational learning (Mineka and Zinbarg, 2006; Field and Purkis, 2011). The aforementioned shift to performance-based goals in secondary education, heightened teacher expectations, and lower teacher support may be associated with more verbal threat information (“if you don’t perform well, you won’t get a good job”), more direct negative experiences (e.g., being told off, or social humiliation, when performance is below what is expected), and more observational threat learning (observing others being told off or humiliated when they perform below expectations). Research supports the idea of performance-goal structures creating anxiety: goal structure has been linked to maths anxiety (Federici et al., 2015; Skaalvik et al., 2017) and Baudoin and Galand (2017) reported that performance-based goal structures were associated with feelings of shame and anxiety in schoolchildren. These heightened expectations and associated threat messages at school may be mirrored in the home environment, and may be exacerbated by certain parenting styles known to increase anxiety, such as over-critical parenting (Creswell et al., 2010).

Given that there is a theoretical route through which the transition could create anxiety, then we can look at the effect that this anxiety might have on the individual-level constructs. Heightened anxiety is associated with patterns of information processing (Hadwin and Field, 2010) such as a tendency to interpret ambiguity in a threatening way (*interpretation bias*) and a tendency to attend to threat in the environment (*attentional bias to threat*). As such, once anxiety is heightened students may attend more to both negative feedback about performance (which may negatively affect learning schema and academic self-concept) and negative social cues (which will affect social schema). Equally, ambiguity about academic and social matters may be interpreted more negatively. In short, the knock-on effect of a transition that creates anxiety will be a processing style that is likely to impact social schema, learning schema, and academic self-concept. Those already prone to anxiety are most likely to experience more anxiety, and greater biases in their information processing, which would lead to more anxiety. This idea is supported by Lester et al. (unpublished) who found that children with greater interpretation bias toward threat experienced higher levels of anxiety before transitioning to secondary education. Of course, this theory also explains variance in the levels of anxiety following the transition (e.g., Grills-Taquechel et al., 2010; Madjar et al., 2016; Lester et al., unpublished), because there will be variance in the negativity in secondary environments and variance in children’s risk for anxiety.

For children for whom the transition creates anxiety (and shifts in their schema and information processing), the attentional control theory, which suggests that anxiety impairs goal-directed attentional systems, offers a theoretical mechanism for why academic performance would be affected (Eysenck et al., 2007). One key assumption of this theory is that anxiety increases the attentional allocation toward threat-related stimuli including both external and internal stimuli (i.e., worrisome thoughts). This theory can explain why the anxiety-inducing transition impairs academic outcomes by reducing the attentional capacity available for cognitive tasks. For example, a child experiencing anxiety because of the transition will allocate less attention to tasks in the lesson, and more attention toward worrisome thoughts, and as a result will perform worse in class. This model has been supported by research conducted on maths anxiety and performance (e.g., Carey et al., 2017). Maths performance is arguably more affected by anxiety as it requires significant executive function skills (Cragg and Gilmore, 2014), and as anxiety takes up the allocation available, the executive function systems required to perform maths tasks efficiently are put under strain and performance on the task diminishes, resulting in poorer academic achievement. The attentional control theory also supports the links found between emotional well-being and academic performance.

The theories of anxiety we have used are illustrative of how psychological theory can and, probably, should be used to try to construct parsimonious theoretical frameworks for the effects of the transition to secondary education. Of course, the theories we chose offer little explanation of depressive symptoms, conduct problems and so on. The point is simply that it is possible to build on well-established psychological theory to explain the interplay between the constructs reviewed in this paper.

### Implications for School Intervention Strategies

The above findings have implications for school-based intervention programs that target the primary-to-secondary education transition. At present, these programs are free to vary between schools and districts and often attempt to improve self-confidence and problem-solving (e.g., Shepherd and Roker, 2005). To date, a few studies have examined the impact of different school programs over the transition to investigate whether this makes the transfer easier for students. One study found systemic strategies (e.g., group work on projects with future classmates and modules taught continuously over the transition) were associated with lower school anxiety, though only in typically developing children (Neal et al., 2016). In addition, Rosenblatt and Elias (2008) found that U.S. children who took part in an intervention focusing on social-emotional learning before transitioning had a smaller decline in GPA when receiving higher dosages of the intervention compared to a low dosage group. Furthermore, Shepherd and Roker (2005) investigated a project run after-school which aimed to build self-esteem and resilience in particularly withdrawn and shy children and found improvements in both self-esteem and social skills and fewer school concerns.

While several interventions focus on social-emotional development and improving social skills, only a small number of studies have investigated ways to improve educational achievement. One study conducted by Siddiqui et al. (2016) evaluated a reading program following the transition that was undertaken by pupils who had not reached the expected level for English in their final year of primary education. Their results found that children receiving the intervention had higher reading scores compared to a control group. However, in an earlier study, Siddiqui et al. (2014) evaluated a summer school program that focused on literacy and numeracy skills and found it was not effective in improving the educational achievement of “at-risk” students.

While it is clear some of these programs have benefits for students, much more research is needed to assess the effectiveness of different types of intervention programs with greater sample sizes and longitudinal investigation. Based on the evidence presented, future programs should focus on increasing perceived social support (including that given by parents, teachers, and peers), continuation of academic study, such as introducing topics in the final year of primary education that are continued through to secondary education, and developing social-emotional interventions that can be administered nationwide which are effective for typically developing children and those with SEN. In addition, the difficulties associated with the transitional period could be eased by preparing children for the change in goal structure, or by secondary schools adopting a mastery environment, which is arguably more beneficial for children’s learning (Meece et al., 2006).

### Limitations

Many of the studies presented here were conducted several years ago, often using longitudinal data collected years prior, which may decrease the relevance to education and schools today. This limitation highlights the need for more research to examine how the changes in our educational systems and the advancements made particularly in schooling and technology may impact both the well-being of students and their learning environment during the transition to secondary education. For example, in comparison to transitional students that participated in studies conducted in the early 2000s, it is now the norm for adolescents to own, or have access to a mobile phone, computer, or tablet (Ofcom, 2017). To date, very few studies have investigated the use of the internet in transition interventions (e.g., Maher, 2010), and it appears there is a lack of investigation into the effects of technology on well-being and achievement during the transitional years. Current investigation into this area is important for a number of reasons. One example to illustrate this is bullying. Advancements in technology have made bullying an online activity resulting in around 49% of children being victims of cyberbullying (Raskauskas and Stoltz, 2007). As discussed previously, the transition may help victimized children renegotiate their social network to no longer be victims of bullying (e.g., Shell et al., 2014; Wang et al., 2016). However, it could be speculated that if evidence of the bullying that occurred during primary education was online, the renegotiation of their social network would not be able to occur, and as such, victimized children would continue to experience bullying throughout secondary education. Another example is the use of the internet to facilitate the transition in a practical manner, i.e., showing interactive maps of the school environment and classrooms to prospective students. An online, interactive map may be beneficial as one of the worries reported by transitional students was being lost (Zeedyk et al., 2003). Another potential use of technology is inviting classroom peers to get to know each other by using a monitored forum where children may introduce themselves and ask their new teacher and a few current students any questions they may have. Additional homework to be completed over the summer prior to the transition could be made available on such a forum to help decrease the interruption in achievement found during the transitional years (Akos et al., 2015). While the above points made are based on speculation alone, future research examining some of these ideas could provide interesting insights into the use of technology during the transition year to help facilitate a successful transition in terms of student well-being, social interaction, academic growth, and environmental practicalities.

Another limitation is the lack of cultural diversity within the research discussed. The studies presented here have focused on particularly “western systems” such as those in America, Europe and Australia. One reason for the lack of diversity is the differences found in systems around the world. For example, children attending schools in places such as Mexico, Africa, or the Middle East, are less likely to attend school past the age of 11 (UNESCO Institute for Statistics, 2010; Gibbs and Heaton, 2014), therefore making the secondary education transition a non-event. However, one of the Sustainable Development Goals set out to achieve by 2030 by the United Nations is to ensure all children complete primary and secondary education. Hopefully, this increase in attendance worldwide allows for comparisons of the transition to be made in the future, allowing for research from a range of culturally diverse countries to examine the issues children encounter during this time, and whether they differ between nations.

## Conclusion

The importance of emotional well-being, specifically within schools, has been acknowledged by the British Psychological Society in a recent briefing paper (BPS, 2017). It is reported that one in four children and adolescents display signs of a mental health difficulty, with up to three children in every classroom experiencing a mental health issue that can be treated. Furthermore, only 25–40% of the young people affected by these issues receive support from a mental health professional early enough in their development, if they receive any help at all. As most of the disorders experienced in childhood and adolescence continue into adulthood, it is beneficial for everyone involved to ensure interventions are administered as early as possible not only for better emotional well-being but also because of the associations with academic achievement and social functioning, all of which are important for a successful transition. To help during this time, parents and schools could aim to provide more social support during and following the transition to increase the perceived support felt by adolescents. Schools could also provide transition strategies that focus on the worries of children such as being lost or being bullied. In addition, schools could teach topics that can be carried on from primary to secondary education to help with the interruption of achievement. Furthermore, children with SEN should have additional support and provisions in place to ensure they transition with as few difficulties as possible.

Despite all the evidence presented, there are still gaps in the literature. The research investigating internalizing and externalizing disorders is particularly sparse and should be the focus for future exploration. Furthermore, researchers should pilot transition strategies in schools based on the recommendations above. Additional research should aim to utilize longitudinal designs measuring a wide range of factors to accurately assess the impact of the school transition on several outcomes including academic achievement and emotional well-being. Hopefully, future research will overcome the inconsistent findings to date and will reliably identify factors that ensure children become well-adjusted to their new environment. By identifying predictive factors of importance for the primary-to-secondary school transition, researchers can help enable every child to have the opportunity to make a successful transition to secondary education and continue to develop academically, socially, and emotionally.

## Author Contributions

All authors contributed to the inception of the paper. DE took the lead on the writing overall (notably the sections “Introduction,” “Discussion,” and the section “The Effects of the Transition to Secondary Education on Emotional Health”), did the initial planning, and coordinated the authors. GB took the lead on writing the section “The Effects of the Transition to Secondary Education on Academic Achievement” and had input on all drafts. AF supervised the project, commented on and edited the first submission, and restructured and edited the revised version by introducing the conceptual framework in **Figure 1**.

## Conflict of Interest Statement

The authors declare that the research was conducted in the absence of any commercial or financial relationships that could be construed as a potential conflict of interest.
